# The post-pandemic library handbook

**DOI:** 10.29173/jchla29786

**Published:** 2024-08-01

**Authors:** Siu Hong Yu

**Affiliations:** Science and Engineering, Librarian University of Waterloo, Waterloo, ON, Canada

Todaro J. **The post-pandemic library handbook**. 1st ed. Lanham: Rowman & Littlefield; 2022. Hardcover: 184 p. ISBN: 978-1-5381-5374-1. Price: USD$95.00. Available from: https://rowman.com/ISBN/9781538153741/The-Post-Pandemic-Library-Handbook

The COVID-19 pandemic profoundly disrupted every aspect of modern society and impacted libraries of all types around the world. From sudden closures and social distancing mandates to the rapid shift towards digital collections and services, library leaders and professionals had to adapt to rapidly changing community and institutional needs by quickly reconfiguring work environments, reassessing collection priorities, and reimagining user engagement [1-4]. The significant increase in demand for COVID-19 literature searches, systematic reviews and data management support from clinicians and researchers also posed unique challenges for medical and academic health sciences libraries to safely operate under pandemic conditions [5,6].

*The Post-Pandemic Library Handbook* was published in 2022 and is one of the earliest comprehensive guides that addresses the emerging post-pandemic best practices for libraries. With American library managers and administrators as its main target audience, the handbook provides a holistic examination of various aspects of library operations, services and management, emphasizing the need for flexibility, innovation and resilience. It also offers actionable guidance and practical strategies for library professionals to navigate the evolving post-pandemic work environments.

Author Dr. Julie Todaro has extensive experience in library leadership, management, and emergency preparedness. She is currently the Dean of Library Services at Austin Community College in Austin, Texas where she manages close to 200 employees across eleven campus libraries in eight counties. With an emphasis on using evidence-based decision-making practices, her library professional career spans over three decades. Todaro is the author of the two editions of *Emergency Preparedness for Libraries* (1st in 2009 and 2nd in 2020), the 2015 *Mentoring A-Z*, and the 2014 *Library Management for the Digital Age: A New Paradigm*. She also co-authored the 2006 *Training Library Staff and Volunteers to Provide Extraordinary Customer Service*. Todaro is a frequent keynote speaker and presenter on the topics of organizational structure design, change management, strategic planning, public relations and marketing, and customer service assessment and accountability.

The book consists of a dedication, preface, introduction, 12 chapters, three appendices, an index, and notes about the author. Chapter 1 has 22 pages and is the longest chapter of the book. It lays the foundation of the book by looking back at the pandemic of 1918 and other public health emergencies to explore why libraries did not learn from the past. In outlining the preexisting pain points such as limitation of physical spaces and inadequate wireless hotspots, Todaro highlights the new issues arisen from the COVID-19 pandemic and summarizes the key points of all the subsequent chapters to answer the central question of “What if we had been ready?” Each chapter that follows addresses a key aspect of librarianship under a global health crisis.

Chapter 2 covers the issues of facilities and physical spaces from the perspectives of users and library workers. The chapter offers insightful comments and recommendations on furniture, signage, spacing, cleaning protocols, the problem with “hoteling” work arrangements, and technical considerations on HVAC. It contains detailed tables on public and workspaces with different functions and activities progressing through different stages during and after the pandemic.

Matters regarding collections and resources are discussed in Chapter 3 followed by the topics of assessment and accountability in Chapter 4. Todaro emphasizes the importance of evidence-based decision making in managing “tomorrow’s collections” that are user-centred. She examines the concept of flexibility in the context of change management across all levels of library organization, and provides useful SWOT analysis tables with guiding questions to help managers and administrators with accountability and assessment. Some of the content on library spaces included in Chapter 3, however, could be better discussed and integrated into Chapter 2 on facilities.

Chapters 5 and 6 examine human resources and communication during emergency events, while Chapters 7 and 8 discuss effective management and leadership. Drawing upon her own professional experiences, Todaro offers well-considered insights on successful change management and trust building while balancing users/stakeholders’ needs with library workers’ safety and wellbeing. Leadership during extreme emergencies should focus on meaningful influence and motivation that affect real and intended change. According to Todaro, expert leaders listen and observe closely with intention, and prioritize timely communication and responsive assessment. With only six pages in length, however, Chapter 5 *Human Resources, Critical Training, and Education* is the shortest chapter and could be expanded to offer more concrete mitigations and support on library frontline workers’ emotional strain during a pandemic.

How best to handle mistakes and failures is considered in Chapter 9, which further discusses the strategies on maintaining trust across library users, workers and stakeholders. Chapter 10 explores the issues of service access and delivery, which could be better grouped and addressed along with Chapter 3’s content on collections and resources. In addition, while the pandemic accelerated the digital shift on library collections and services, this handbook should further discuss its implications and offer practical recommendations in addressing the digital divide and equitable access from the library users and workers’ perspectives as recommended from a 2021 OCLC research briefing [3].

With the focus on maintaining libraries’ mission and trust, the discussions on public relations, marketing and branding in Chapter 11 are highly relevant but could be moved and considered along with Chapter 6’s communication content for a better flow. Lastly, Chapter 12 *Returning to Begin Again* reiterates Todaro’s key positive message of reinvention and moving forward. Each chapter ends with a value-added discussion on the respective topics from the smaller and solo libraries’ perspectives.

In terms of supplementary materials, Appendix C *Recommended Resources* provides a list of reputable organizations and content aggregators rather than specific resource items or publications to avoid inactive hyperlinks or being outdated. The Index is also quite comprehensive and granular (COVID is separated into pre-COVID and post-COVID for example) to help with quick information retrieval.

Overall, this authoritative handbook addresses the knowledge gaps at the time when the world was dealing with the “new normal” *post*-pandemic to ensure business continuity across different libraries of all types and sizes. Despite some shortcomings, this is a valuable resource for library administrators and professionals to prepare for the next pandemic.
